# Recent alcohol consumption and risk of incident ovarian carcinoma: a pooled analysis of 5,342 cases and 10,358 controls from the Ovarian Cancer Association Consortium

**DOI:** 10.1186/1471-2407-13-28

**Published:** 2013-01-22

**Authors:** Linda E Kelemen, Elisa V Bandera, Kathryn L Terry, Mary Anne Rossing, Louise A Brinton, Jennifer A Doherty, Roberta B Ness, Susanne Krüger Kjær, Jenny Chang-Claude, Martin Köbel, Galina Lurie, Pamela J Thompson, Michael E Carney, Kirsten Moysich, Robert Edwards, Clare Bunker, Allan Jensen, Estrid Høgdall, Daniel W Cramer, Allison F Vitonis, Sara H Olson, Melony King, Urmila Chandran, Jolanta Lissowska, Montserrat Garcia-Closas, Hannah Yang, Penelope M Webb, Joellen M Schildkraut, Marc T Goodman, Harvey A Risch

**Affiliations:** 1 Department of Population Health Research, Alberta Health Services-Cancer Care and Departments of Medical Genetics and Oncology, University of Calgary, Calgary, AB, Canada; 2 The Cancer Institute of New Jersey, Robert Wood Johnson Medical School, New Brunswick, NJ, USA; 3 Obstetrics and Gynecology Epidemiology Center, Brigham and Women’s Hospital, Boston, MA, USA; 4 Department of Epidemiology, Harvard School of Public Health, Boston, MA, USA; 5 Fred Hutchinson Cancer Research Center, Seattle, WA, USA; 6 Division of Cancer Epidemiology and Genetics, National Cancer Institute, Bethesda, MD, USA; 7 University of Texas School of Public Health, Houston, TX, USA; 8 Danish Cancer Society Research Center, Copenhagen, Denmark; 9 Gynecologic Clinic, Rigshospitalet, University of Copenhagen, Copenhagen, Denmark; 10 Division of Cancer Epidemiology, German Cancer Research Center, Heidelberg, Germany; 11 Department of Pathology and Laboratory Medicine, Calgary Laboratory Services, Calgary, AB, Canada; 12 Cancer Research Center, University of Hawaii, Honolulu, HI, USA; 13 Roswell Park Cancer Center, Buffalo, NY, USA; 14 Magee Womens Research Institute, Pittsburgh, PA, USA; 15 University of Pittsburgh School of Public Health, Pittsburgh, PA, USA; 16 Memorial Sloan-Kettering Cancer Center, New York, NY, USA; 17 Department of Cancer Epidemiology and Prevention, The M. Sklodowska-Curie Cancer Center and Institute of Oncology, Gliwice, Poland; 18 Division of Genetics and Epidemiology, Institute of Cancer Research, Sutton, United Kingdom; 19 The Queensland Institute of Medical Research, Locked Bag 2000 Royal Brisbane Hospital, Herston, Australia; 20 Department of Community and Family Medicine and the Comprehensive Cancer Center, Duke University Medical Center, Durham, NC, USA; 21 Departments of Medicine and Biomedical Sciences, Cedars-Sinai Medical Center, Los Angeles, CA, USA; 22 Department of Chronic Disease Epidemiology, Yale School of Public Health, New Haven, CT, USA

## Abstract

**Background:**

Studies evaluating the association between alcohol intake and ovarian carcinoma (OC) are inconsistent. Because OC and ovarian borderline tumor histologic types differ genetically, molecularly and clinically, large numbers are needed to estimate risk associations.

**Methods:**

We pooled data from 12 case-control studies in the Ovarian Cancer Association Consortium comprising 5,342 OC cases, 1,455 borderline tumors and 10,358 controls with quantitative information on recent alcohol intake to estimate odds ratios (OR) and 95% confidence intervals (CI) according to frequencies of average daily intakes of beer, wine, liquor and total alcohol.

**Results:**

Total alcohol intake was not associated with all OC: consumption of >3 drinks per day compared to none, OR=0.92, 95% CI=0.76-1.10, P trend=0.27. Among beverage types, a statistically non-significant decreased risk was observed among women who consumed >8 oz/d of wine compared to none (OR=0.83, 95% CI=0.68-1.01, P trend=0.08). This association was more apparent among women with clear cell OC (OR, 0.43; 95% CI, 0.22-0.83; P trend=0.02), although based on only 10 cases and not statistically different from the other histologic types (P value for statistical heterogeneity between histologic types = 0.09). Statistical heterogeneity of the alcohol- and wine-OC associations was seen among three European studies, but not among eight North American studies. No statistically significant associations were observed in separate analyses evaluating risk with borderline tumors of serous or mucinous histology. Smoking status did not significantly modify any of the associations.

**Conclusions:**

We found no evidence that recent moderate alcohol drinking is associated with increased risk for overall OC, or that variation in risk is associated strongly with specific histologic types. Understanding modifiable causes of these elusive and deadly cancers remains a priority for the research community.

## Background

Carcinomas classified as ovarian are the fourth most common female cancer, accounting for 225,000 (3.7%) of all new cases and 140,000 (4.2%) of all cancer deaths globally [1]. Known mutations in high penetrance genes are the best-defined risk factors, explaining ~10-15% of all epithelial ovarian carcinomas [2-6], while common variants in low penetrance genes may account for a smaller fraction (~3%) of the polygenic component [7-9]. Non-genetic factors associated with the development of ovarian carcinoma include reduced risk with oral contraceptive use [10,11], number of full-term pregnancies [12,13], long-term breastfeeding [14] and tubal ligation or salpingectomy in *BRCA1/2* mutation carriers [12]. The independent contribution of modifiable environmental [15,16] and lifestyle or behavioral [17-21] factors including diet is inconclusive, and only a few studies have confirmed non-genetic risk factor associations according to histologic type [14,22-25].

Several studies examined the association between total alcohol consumption and ovarian carcinoma and reported inverse [17,26,27], null [28-31], or positive [32,33] trends with the highest category of alcohol intake. Increased risk was also found among the mucinous histologic type [34,35]. An earlier pooled analysis of prospective studies found no association between ≥30 g/d total alcohol intake compared to 0 g/d among 2,001 cases of ovarian carcinoma (RR, 1.12; 95% CI, 0.86-1.44), or for alcohol modeled continuously among 121 cases with mucinous histology (RR, 1.06; 95% CI, 0.84-1.34) [36]. A previous meta-analysis reported no overall association between alcohol consumption and ovarian carcinoma, but did find a 6% increased risk of mucinous ovarian carcinomas (95% CI, 1.01, 1.12, n=581) with each increase in intake of 10 g/day alcohol using continuous estimates obtained from authors of primary reports [37]. A more recent meta-analysis of 27 observational studies found no overall association of moderate or heavy drinking, but found an inverse trend with endometrioid ovarian carcinoma from three studies reporting associations by histology [38]. Two other reports summarized the epidemiologic evidence of the relation between alcohol and ovarian carcinoma descriptively [39] and as a systematic review [27]. Reviews or meta-analytic techniques that summarize categorical data from primary investigations comparing highest to lowest intakes have several limitations, including a loss of data when intermediate intake categories are excluded, which may introduce reporting bias, a problem termed “publication bias *in situ*” [40]. Additionally, primary studies differ in their adjustment for important confounders, in whether they distinguish invasive cancers from borderline tumors, which differ genetically, molecularly and clinically [41,42], and in whether they reported associations separately by histologic type. These differences challenge the ability to synthesize published findings. To circumvent these limitations, we conducted a large pooled analysis of original data from 12 studies participating in the Ovarian Cancer Association Consortium (OCAC).

## Methods

### Study subjects

Twelve studies of ovarian cancer that contributed data are described in Table 1. All studies used population-based ascertainment methods for identifying eligible cases and controls and most studies matched cases to controls on age or age and region of residence. Eight studies were from the United States or Canada (CON [43], DOV [44], HAW [45], HOP [46], NCO [47,48], NEC [49,50], NJO [51,52] and SON [53]), three were from Europe (GER [54], MAL [55-57] and POL [58]) and one was from Australia (AUS [59]). Informed consent was obtained from participating subjects in each of the individual studies, and local human research investigations committees approved each study.

**Table 1:** Overview of OCAC studies

**Study acronym**	**Study name**	**Controls, n**	**Cases, n**						White non-Hispanic %^*^	**Carcinoma cases with grade information %**†	**Recruitment year and location**	**Matching variables**‡
			**Border-line**	**All carcinomas§**	**Serous**	**Muc-inous**	**Endo-metrioid**	**Clear Cell**				
AUS [59]	AOCS (Australian Ovarian Cancer Study) and ACS (Australian Cancer Study – Ovarian Cancer)	1,333	259	882	537	39	106	71	93.3	92.8	2002-2006; Australia	State of residence and 5-year age groups
CON [43]	CON (Connecticut Ovarian Cancer Study)	526	103	339	193	18	70	33	91.7	85.4	1998-2003; Connecticut, USA	3 age strata (35-49, 50-64 and 65-79 years)
DOV [44]	DOVE (Diseases of the Ovary and their Evaluation)	1,116	189	483	269	20	81	31	90.9	82.7	2002-2005 and 2006-2009; Washington, USA	5-year age groups, 1-year calendar intervals and two county strata
GER [54]	GOCS (German Ovarian Cancer Study)	502	30	209	107	24	23	6	99.9	100	1993-1996; Germany	Age and study region
HAW [45]	HAWAII (Hawaii Ovarian Cancer Study)	1,100	97	384	176	42	68	50	31.9	91.3	1993-2008; Hawaii, USA	5-year age groups and race
HOP [46]	HOPE (Hormones and Ovarian Cancer Prediction Study)	1,365	76	530	289	27	71	46	96.0	94.1	2003-2009; Pennsylvania, USA	5-year age groups and area code plus 3 number prefix
MAL [55-57]	MALOVA (Malignant Ovarian Cancer Study)	908	115	267	157	30	41	21	100	93.9	1994-1999; Denmark	5-year age groups
NCO [47,48]	NCOCS (North Carolina Ovarian Cancer Study)	979	212	777	429	44	126	82	80.9	100	1999-2008; North Carolina, USA	5-year age groups and race
NEC [49,50]	NECC (New England-based Case-Control Study)	1,109	274	707	386	47	152	96	96.3	100	1992-1997 and 1998-2003; New England, USA	5-year age groups and region of residence
NJO [51,52]	NJOCS (New Jersey Ovarian Cancer Study)	277	0	183	104	7	30	24	87.6	87.2	2002-2008; New Jersey, USA	None
POL [58]	POL (Polish Ovarian Cancer Control Study)	601	18	236	101	25	52	13	100	66.3	2000-2003; Poland	5-year age groups and study center
SON [53]	SON (Southern Ontario Study of Reproduction, Diet and Health)	542	82	345	200	38	65	28	98.3	0	1989-1992; Southern Ontario	3 age strata (35-49, 50-64 and 65-79 years)
Totals		10,358	1,455	5,342	2,948	361	885	501	87.7	84.86		

* White non-Hispanic subjects as a percentage of all race-ethnicities enrolled in each study.

† Percentages reflect grade available for serous, mucinous and endometrioid carcinomas and for which we applied the algorithm to reduce histologic misclassification (see Methods).

‡ All studies except GER used frequency matching.

§ Includes the epithelial histologic types: serous, mucinous, endometrioid, clear cell, mixed epithelial, transitional cell, squamous cell, and undifferentiated.

### Alcohol assessment and covariate data collection

The unit of analysis for alcohol consumption was average daily grams of alcohol intake (g/d). Daily alcohol intake was estimated using validated food frequency questionnaires (FFQs) in AUS [60], DOV [61], HAW [62], MAL, NEC [63], NJO [51] and SON [53]. The exposure period was the year preceding recruitment (AUS, HAW, MAL, NEC, NJO and SON) or the time period approximately four years before the reference date (DOV). The remaining studies did not use FFQs but embedded questions regarding alcohol intake in risk factor questionnaires (CON [43], GER, HOP [34], NCO and POL). The exposure period for these studies was habitual regular drinking at the reference date (HOP) or the time period approximately five years before the reference date (CON, GER, NCO and POL). Daily alcohol intake for all studies was calculated by summing the product of the frequency of consumption of a specified serving of alcoholic beverage (beer, wine and liquor) by the alcohol content of that beverage using national estimates of alcohol content for that country. Total alcohol was estimated as the sum of alcohol intake across all alcoholic beverage types and submitted for pooled analysis. A subset of studies (AUS, CON, DOV, HAW, HOP and NEC) provided information for white and red wine separately.

Key clinical, demographic and questionnaire data on study subjects were merged into a common dataset and included case-control status, ethnicity/race, tumor behavior and histology, age at diagnosis (or comparable reference date for controls), history of prior cancers, current/former/never smoking status, menopausal status, oral contraceptive use, tubal ligation, endometriosis, hysterectomy, family history of breast or ovarian cancer in first-degree relatives, parity, age at last parturition, interview year, age at menarche, body mass index (BMI) and study site. Total energy intake was obtained from studies that collected dietary information using FFQs (AUS, DOV, HAW, NEC, NJO and SON). The data were checked for consistency and completeness and discrepancies were followed-up with individual study investigators.

We excluded from analyses subjects with non-epithelial ovarian tumors, prior histories of cancer other than non-melanoma skin cancer or subjects with missing information for total alcohol intake. Data were available from 5,342 cases of incident ovarian carcinoma, 1,455 women with incident ovarian borderline tumors and 10,358 controls (Table 1).

### Statistical analysis

The studies were combined into a single dataset for analysis. Alcohol intake categories were derived in increments of one standard drink (g ethanol content) consumed daily: alcohol from any source (10 g); 12 oz beer (12.2 g), 4 oz wine (10.5 g) and 1 oz liquor (9.5 g). Primary analyses evaluated associations between alcohol intake and risk of ovarian carcinoma (excluding borderline tumors) using unconditional logistic regression to estimate odds ratios (OR) and 95% confidence intervals (CI). Trends in risk were evaluated by modeling the ordinal variable representing the category values of alcohol intake (e.g., 1, 2, 3) in the regression models with 1 degree-of-freedom [64]. Statistical heterogeneity in ORs across studies was evaluated using the likelihood ratio test comparing models with and without an interaction term between alcohol intake and study site. To describe further the degree of statistical heterogeneity, we estimated *I*^*2*^, the between-group variance [65], which describes the proportion of total variation in estimates of the ORs due to the heterogeneity between groups of studies. We estimated *I*^*2*^ to evaluate statistical heterogeneity between studies defined by their continent of origin. Groups of studies with statistically homogeneous ORs have an *I*^*2*^ value of zero.

All models were adjusted for the known or potential confounders footnoted in the tables. Risk models associated with total alcohol intake did not include other alcoholic beverage types. Risk models associated with beer, wine or liquor intake included all three beverage types and were thus adjusted for each other. Risk models associated with white or red wine intake included both types of wine as well as beer and liquor intake. To account for potential heterogeneity of summary risk estimates across studies, all models included interaction terms between every non-alcohol covariate and study site and are thus equivalent to fixed-effects meta-analyses, although the exclusion of these terms did not alter the risk estimates appreciably (data not shown). In addition, among a subset of studies, primary analyses were also adjusted for total energy intake, excluding subjects with extreme total energy values as previously described [66] and using the residual method [67], in order to evaluate the extent of confounding from this variable.

For the 12 studies combined, we simultaneously modeled the risk of each of five histologic types of ovarian carcinoma (high-grade serous, low-grade serous, mucinous, endometrioid and clear cell) and two of the four main types of borderline tumors with sufficient numbers for analysis (serous and mucinous) using polytomous logistic regression [68]. Risk models were adjusted for all covariates but excluded the interactions between non-alcohol covariates and study site to ease statistical computation. Statistical heterogeneity of the alcohol-ovarian tumor histology associations was tested separately for the carcinomas and the borderline tumors and was evaluated using the type 3 analysis of effects with degrees-of-freedom equal to the number of response levels minus one times the number of exposure levels minus one [68]. For these models, we incorporated considerations from the contemporary pathology literature to refine risk associations in the analyses of histologic type, as implemented previously [69]. Specifically, others have shown that an appreciable proportion of grade 3 mucinous ovarian carcinomas are, in fact, metastatic from the gastrointestinal tract [70], up to one-third of endometrioid ovarian carcinomas are high-grade serous ovarian carcinomas [71,72] and approximately 3% of epithelial ovarian carcinomas are low-grade serous [71,72]. We, therefore, re-assigned histologic type according to the expected distributions of histology combined with grade observed from large population-based series [71,72] as follows. Endometrioid carcinomas were re-classified as high-grade serous carcinomas if their grade was ≥G3, mucinous carcinomas were assumed to be metastatic and excluded from analysis if ≥G3, and serous histology was re-classified as either low-grade serous carcinomas (G1) or high-grade serous carcinomas (≥G2).

Because of the reported association between smoking and ovarian carcinoma and, particularly for mucinous ovarian carcinoma and mucinous borderline tumors [22,24,25], statistical interaction was evaluated using the likelihood ratio test comparing models with and without an interaction term for the categorical forms of alcohol intake and smoking status (never, current and former). We also performed stratified analyses of alcohol intake across categories of smoking status. Potential modification of the alcohol-ovarian carcinoma association by other variables was examined using a similar approach.

Statistical tests were two-sided and implemented with SAS (SAS Institute, Cary, NC, Version 9.1). Funnel plots representing the study-specific and combined data estimates were derived from the logistic regression models as described above.

## Results

### Characteristics of included studies and participants

Table 1 describes the characteristics of the 12 case-control studies. Eighty-eight percent of the cases were white non-Hispanic and ~85% of the carcinomas had information on tumor grade. The distribution of alcohol intake is shown in Additional file 1: Table S1, overall and for each study separately. Overall, average daily total alcohol intake ranged from approximately one-fifth of a standard drink at the 25^th^ percentile to 1-2 drinks at the 75^th^ percentile of the distributions. More women consumed wine than the other alcoholic beverages.

Generally, cases and controls were similar in their distributions across covariates (Additional file 1: Table S2**).** As expected, however, cases were more frequently postmenopausal than controls, were more likely to be nulliparous, and less likely than controls to have used oral contraceptive hormones for appreciable durations or to have had tubal ligation or a hysterectomy. The majority (~75%) of subjects were recruited in the past decade. The distribution of covariates did not differ by much among controls who consumed beer, wine or liquor except, perhaps, that fewer wine consumers were current smokers and a greater proportion of beer drinkers were pre- or peri-menopausal.

### Alcohol consumption and risk of ovarian carcinoma

**Figure 1:**
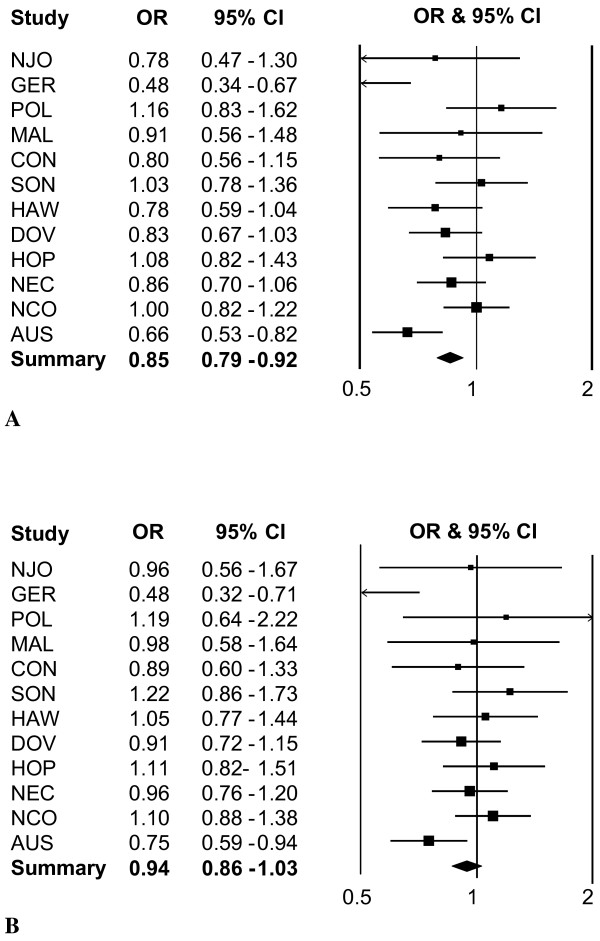
**Funnel plot of study-specific and summary OR and 95% CI for the association between alcohol intake (none, any) and ovarian carcinoma in 12 OCAC studies. **Squares indicate study-specific OR; the size of the squares is proportional to study-specific sample size; the width of lines indicates the study-specific 95% CI; diamonds indicate summary OR; the width of the diamonds indicates summary 95% CI. Refer to Table 1 for study nomenclature. 1A**: **Age and race adjusted OR and 95% CI. Statistical heterogeneity in ORs across studies, P value < 0.0001 (see Statistical analysis). 1**B:** Multivariable-adjusted OR and 95% CI. Adjusted for variables in footnote of Table 2. Statistical heterogeneity in ORs across studies, P value = 0.03.

In multivariable-adjusted pooled analyses, total alcohol intake from any source was not associated with risk of ovarian carcinoma (consumption of >3 drinks per day compared to none: OR=0.92, 95% CI=0.76-1.10, P trend=0.27; Table 2). Given the absence of a dose-response relationship, we modeled the variable dichotomously (none, any regular consumption) (Figure 1). Adjustment for known or suspected confounders beyond age and race (Figure 1A**)** tended to attenuate risk associations (Figure 1B) indicating the importance of accounting for these variables in the analysis. Further adjustment for total energy had little effect (data not shown).

**Table 2:** Association between consumers of alcoholic beverages and ovarian carcinoma, OCAC studies

**Intake/d**	**Ca/Co**	**OR (95% CI)**
**Total alcohol**	**5,342/10,358**	
None	2,269/4,296	1.0 (Ref)
Up to 1 drink *	2,074/3,928	0.94 (0.85-1.03)
1-2 drinks	560/1,112	0.97 (0.85-1.11)
2-3 drinks	192/400	0.91 (0.74-1.11)
>3 drinks	247/622	0.92 (0.76-1.10)
P trend		0.27
**Beer**†		
None	4,016/7,472	1.0 (Ref)
Up to 12 oz	1,179/2,570	0.92 (0.83-1.02)
>12 oz	147/315	1.09 (0.86-1.37)
P trend		0.43
**Wine**†		
None	2,821/5,307	1.0 (Ref)
Up to 4 oz	2,057/3,984	0.94 (0.85-1.04)
4-8 oz	261/522	1.00 (0.83-1.20)
>8 oz	203/545	0.83 (0.68-1.01)
P trend		0.08
**White wine**‡		
None	2,110/4,114	1.0 (Ref)
Up to 4 oz	1,053/2,032	0.93 (0.82-1.06)
>4 oz	162/406	0.97 (0.77-1.21)
P trend		0.26
**Red wine**‡		
None	2,330/4,548	1.0 (Ref)
Up to 4 oz	866/1,665	0.92 (0.81-1.05)
>4 oz	129/336	0.90 (0.71-1.15)
P trend		0.11
**Liquor**†		
None	3,599/6,865	1.0 (Ref)
Up to 1 oz	1,535/3,061	0.97 (0.88-1.08)
>1 oz	208/431	1.03 (0.85-1.26)
P trend		0.82

Adjusted for age (<40; 40-49; 50-59; 60-69; 70+ years), smoking status (never, former, current), site (AUS, CON, DOV, GER, HAW, HOP, MAL, NCO, NEC, NJO, POL, SON), race/ethnicity (white nonHispanic; white Hispanic; black non Hispanic; Asian; other or unknown); menopausal status (pre/peri-menopausal; postmenopausal, unknown or missing), oral contraceptive use (<6mo, 6-22 mo, 23+ mo, unknown or missing), tubal ligation (yes; no; unknown or missing), endometriosis (yes; no; unknown or missing), hysterectomy (yes; no; unknown or missing), family history of breast or ovarian cancer in first-degree relatives (no; yes; unknown; no daughters or sisters), parity/age at last birth (nulliparous; 1-2 births/age ≤25 yrs at last pregnancy; 3+ births/age ≤25 yrs at last pregnancy; 1-2 births/age >25 years at last pregnancy; 3+ births/age >25 years at last pregnancy; yes if ever pregnant but unknown or missing age at last pregnancy age; no or unknown if ever pregnant and missing age at last pregnancy, interview year (1990-1994; 1995-1999; 2000-2004; 2005-2009; missing), age at menarche (8-10 yrs; 11 yrs; 12 yrs; 13 yrs; 14-21 yrs; <8 or ≥ 22 yrs), body mass index (continuous) and education (less than high school, high school, some college, completed college or university, completed graduate or professional degree, missing). Models include interaction terms between site and each covariate except alcohol.

* 1 drink = 10 grams ethanol.

† Models are also simultaneously adjusted for consumption of beer, wine and liquor intake.

‡ White/red wine information available from AUS, CON, DOV, HAW, HOP and NEC only. Models are simultaneously adjusted for beer and liquor intake.

### Alcoholic beverage type and risk of ovarian carcinoma

All studies provided information on type of alcoholic beverage consumed (beer, wine and liquor). Compared to women who reported no wine intake, we observed a statistically non-significant decreased risk associated with consumption of more than 8 oz/d of wine after adjusting for consumption of other types of alcoholic beverage intake (OR=0.83, 95% CI=0.68-1.01, P trend=0.08; Table 2). Associations did not differ by much when we restricted the analyses to those individuals who consumed only one type of alcoholic beverage (data not shown). Among a subset of studies with information on white or red wine consumed, risk associations were not statistically significant.

### Alcohol and ovarian tumor histologic types

More than 3 average drinks/d of alcohol intake from any source was associated with a lower risk of endometrioid ovarian carcinoma (OR=0.49, 95% CI=0.27-0.91), although this was no longer evident when the two highest intake categories were combined (>2 drinks/d: OR=0.85, 95% CI=0.58-1.26; P trend=0.45; Table 3). We observed a statistically significant inverse trend between consumption of wine and clear cell ovarian carcinomas with a decreased risk at higher intakes only (>8 oz/d: OR=0.43, 95% CI=0.22-0.83; P trend=0.02). This association, however, was based on 10 cases and the heterogeneity between histologic types was not statistically significant (P heterogeneity=0.09). Following combining the two highest intake categories, the association remained suggestive (>4 oz/d: OR=0.70, 95% CI=0.47-1.03, P trend=0.05; Table 3). A statistically non-significant increased risk was also seen between total alcohol intake over 3 average drinks/d and mucinous borderline tumors (OR=1.40, 95% CI=0.99-1.20; P trend=0.22, Table 4, data shown for total alcohol and wine only) but disappeared following combining the two highest intake categories (>4 oz/d: OR=1.22, 95% CI=0.90-1.66, P trend=0.42; Table 4). Application of the pathology-based algorithm tended to shift estimates and 95% CIs farther from the null, although there was no appreciable difference in significance of estimates when the algorithm was not implemented (Additional file 1: Table S3).

**Table 3:** Association between total alcohol and wine intake and histological types* of ovarian carcinoma, OCAC studies

**Intake/d**	**Controls N=10,358**	**High-Grade Serous N=2,580**		**Mucinous N=245**		**Endometrioid N=506**		**Clear Cell N=501**		**Low-Grade Serous N=198**		
	**Co**	**Ca**	**OR (95% CI)**	**Ca**	**OR (95% CI)**	**Ca**	**OR (95% CI)**	**Ca**	**OR (95% CI)**	**Ca**	**OR (95% CI)**	***P *value†**
**Total alcohol** ‡												
None	4,296	1,060	1.0 (Ref)	98	1.0 (Ref)	214	1.0 (Ref)	223	1.0 (Ref)	61	1.0 (Ref)	
Up to 1 drink	3,928	1,029	0.95 (0.84-1.07)	97	1.08 (0.76-1.52)	207	0.97 (0.76-1.23)	188	0.84 (0.66-1.07)	90	0.95 (0.65-1.38)	
1-2 drinks	1,112	282	0.97 (0.83-1.15)	26	1.08 (0.66-1.75)	50	0.96 (0.68-1.36)	53	0.97 (0.69-1.37)	29	1.28 (0.78-2.11)	
2-3 drinks	400	79	0.78 (0.60-1.02)	11	1.36 (0.69-2.67)	23	1.36 (0.85-2.19)	17	0.96 (0.56-1.63)	8	0.98 (0.45-2.13)	
>3 drinks	622	130	0.96 (0.77-1.20)	13	0.98 (0.52-1.82)	12	0.49 (0.27-0.91)	20	0.82 (0.50-1.34)	10	1.12 (0.55-2.29)	
P trend			0.31		0.71		0.25		0.50		0.54	0.67
>2 drinks§	1,022	209	0.88 (0.74-1.06)	24	1.12 (0.69-1.83)	35	0.85 (0.58-1.26)	37	0.88 (0.60-1.30)	18	1.05 (0.59-1.86)	
P trend §			0.24		0.62		0.45		0.52		0.53	0.71
**Wine**												
None	5,307	1,316	1.0 (Ref)	128	1.0 (Ref)	263	1.0 (Ref)	272	1.0 (Ref)	81	1.0 (Ref)	
Up to 4 oz	3,984	1,022	0.93 (0.83-1.04)	103	1.12 (0.81-1.54)	206	0.96 (0.77-1.21)	195	0.87 (0.69-1.09)	91	0.94 (0.66-1.34)	
4-8 oz	522	129	0.93 (0.75-1.16)	10	1.03 (0.52-2.04)	22	0.98 (0.61-1.58)	24	0.95 (0.60-1.50)	13	1.33 (0.70-2.50)	
>8 oz	545	113	0.86 (0.68-1.09)	4	0.39 (0.14-1.09)	15	0.68 (0.39-1.20)	10	0.43 (0.22-0.83)	13	1.35 (0.71-2.56)	
P trend			0.13		0.32		0.29		0.02		0.34	0.09
>4 oz §	1,067	242	0.89 (0.75-1.06)	14	0.70 (0.39-1.27)	37	0.84 (0.57-1.23)	34	0.70 (0.47-1.03)	26	1.34 (0.81-2.20)	
P trend §			0.13		0.61		0.41		0.05		0.44	0.25

Adjusted for age (<40; 40-49; 50-59; 60-69; 70+ years), smoking status (never, former, current), site (AUS, CON, DOV, GER, HAW, HOP, MAL, NCO, NEC, NJO, POL, SON), race/ethnicity (white nonHispanic; white Hispanic; black non Hispanic; Asian; other or unknown); menopausal status (pre/peri-menopausal; postmenopausal, unknown or missing), oral contraceptive use (<6mo, 6-22 mo, 23+ mo, unknown or missing), tubal ligation (yes; no; unknown or missing), endometriosis (yes; no; unknown or missing), hysterectomy (yes; no; unknown or missing), family history of breast or ovarian cancer in first-degree relatives (no; yes; unknown; no daughters or sisters), parity/age at last birth (nulliparous; 1-2 births/age ≤25 yrs at last pregnancy; 3+ births/age ≤25 yrs at last pregnancy; 1-2 births/age >25 years at last pregnancy; 3+ births/age >25 years at last pregnancy; yes if ever pregnant but unknown or missing age at last pregnancy age; no or unknown if ever pregnant and missing age at last pregnancy, interview year (1990-1994; 1995-1999; 2000-2004; 2005-2009; missing), age at menarche (8-10 yrs; 11 yrs; 12 yrs; 13 yrs; 14-21 yrs; <8 or ≥ 22 yrs), body mass index (continuous) and education (less than high school, high school, some college, completed college or university, completed graduate or professional degree, missing).

* Cases restricted to samples with information on grade for serous, mucinous and endometrioid histologic types, and for whom we applied the algorithm to reduce histologic misclassification (see Methods).

† *P* for tumor heterogeneity derived from testing the trend variable for alcohol or wine intake in polytomous regression models with 5 df (see Statistical analysis).

‡ 1 drink = 10 grams ethanol.

§ Risk estimates and P trend values are from models that collapse the two highest intake categories.

Models are also simultaneously adjusted for consumption of beer and liquor intake.

**Table 4:** Association between total alcohol and wine intake and ovarian borderline tumors, OCAC studies

**Intake/d**	**Controls N=10,358**	**Serous borderline N=818**		**Mucinous borderline N=561**		
**Total alcohol** *		**Ca**	**OR (95% CI)**	**Ca**	**OR (95% CI)**	***P *value†**
None	4,296	297	1.0 (Ref)	181	1.0 (Ref)	
Up to 1 drink	3,928	349	0.87 (0.72-1.05)	245	0.95 (0.76-1.20)	
1-2 drinks	1,112	86	0.91 (0.69-1.20)	59	0.86 (0.62-1.20)	
2-3 drinks	400	32	1.04 (0.69-1.56)	23	0.94 (0.58-1.51)	
>3 drinks	622	54	1.19 (0.86-1.66)	53	1.40 (0.99-1.20)	
P trend			0.45		0.22	0.39
>2 drinks §	1,022	86	1.13 (0.86-1.49)	76	1.22 (0.90-1.66)	
P trend §			0.63		0.42	0.67
**Wine**						
None	5,307	405	1.0 (Ref)	263	1.0 (Ref)	
Up to 4 oz	3,984	336	0.89 (0.74-1.06)	236	0.89 (0.72-1.10)	
4-8 oz	522	38	0.99 (0.68-1.43)	26	0.88 (0.57-1.36)	
>8 oz	545	39	0.99 (0.69-1.44)	36	1.03 (0.69-1.52)	
P trend			0.66		0.72	0.87
>4 oz §	1,067	77	0.99 (0.75-1.31)	62	0.96 (0.70-1.31)	
P trend §			0.52		0.52	0.69

Adjusted for age (<40; 40-49; 50-59; 60-69; 70+ years), smoking status (never, former, current), site (AUS, CON, DOV, GER, HAW, HOP, MAL, NCO, NEC, NJO, POL, SON), race/ethnicity (white nonHispanic; white Hispanic; black non Hispanic; Asian; other or unknown); menopausal status (pre/peri-menopausal; postmenopausal, unknown or missing), oral contraceptive use (<6mo, 6-22 mo, 23+ mo, unknown or missing), tubal ligation (yes; no; unknown or missing), endometriosis (yes; no; unknown or missing), hysterectomy (yes; no; unknown or missing), family history of breast or ovarian cancer in first-degree relatives (no; yes; unknown; no daughters or sisters), parity/age at last birth (nulliparous; 1-2 births/age ≤25 yrs at last pregnancy; 3+ births/age ≤25 yrs at last pregnancy; 1-2 births/age >25 years at last pregnancy; 3+ births/age >25 years at last pregnancy; yes if ever pregnant but unknown or missing age at last pregnancy age; no or unknown if ever pregnant and missing age at last pregnancy, interview year (1990-1994; 1995-1999; 2000-2004; 2005-2009; missing), age at menarche (8-10 yrs; 11 yrs; 12 yrs; 13 yrs; 14-21 yrs; <8 or ≥ 22 yrs), body mass index (continuous) and education (less than high school, high school, some college, completed college or university, completed graduate or professional degree, missing). Wine consumption was additionally adjusted for other alcoholic beverage types. Models include interaction terms between site and each covariate except alcohol.

* 1 drink = 10 grams ethanol.

† *P *for tumor heterogeneity derived from testing the trend variable for alcohol or wine intake in polytomous regression models with 2 df (see Statistical analysis).

§ Risk estimates and P trend values are from models that collapse the two highest intake categories.

Models are also simultaneously adjusted for consumption of beer and liquor intake.

### Potential sources of effect modification

The association between total alcohol intake (none, any regular consumption) and risk of ovarian carcinoma varied somewhat across studies following multivariable adjustment (Figure 1B**,** P interaction=0.03); the source of heterogeneity was within the three European studies when evaluated by continent of study origin (within-group heterogeneity: Europe, *I*^*2*^=75%; North America, *I*^*2*^=0%). The association between wine intake (none, any regular consumption) and risk of ovarian carcinoma also varied across studies following multivariable adjustment (P interaction=0.01) and significant heterogeneity was again observed within the European studies (*I*^*2*^=81%). Within North American studies, the estimates for wine intake were statistically homogeneous for ovarian carcinoma overall (OR, 0.99; 95% CI, 0.89-1.10; *I*^*2*^=0%). We evaluated whether the decreased risk observed between wine intake and clear cell carcinomas (Table 3) was influenced by the variability within European studies by excluding the three European studies. The association between consumption of >8 oz/d wine and clear cell ovarian carcinomas remained significant (OR=0.48, 95% CI=0.25-0.95; P trend=0.03; 10 cases). Alcohol, in general, has been reported to reduce cellular proliferation by influencing the insulin and insulin-like growth factor (IGF) pathways [73,74], and these pathways have been implicated in the early development and prognosis of clear cell carcinoma types [75-78]. Because obesity is associated with impaired insulin sensitivity [74], we tested the trend association of alcohol or wine intake with histologic types stratified by BMI (<30 vs ≥30 kg/m^2^). There was no clear effect modification by BMI among any of the histologic types for total alcohol intake (data not shown), and suggestive decreased risks for wine intake at lower BMI for both clear cell carcinomas (BMI <30 kg/m^2^: OR=0.83, 95% CI=0.69-1.00; n=382 cases vs BMI ≥30 kg/m^2^: OR=0.79, 95% CI=0.53-1.18; n=119 cases) and high-grade serous carcinomas (BMI <30 kg/m^2^: OR=0.90, 95% CI=0.83-0.98; n=2,052 cases vs BMI ≥30 kg/m^2^: OR=1.17, 95% CI=0.96-1.42; n=528 cases). Smoking status did not significantly modify the association between ovarian carcinoma and total alcohol intake (P interaction=0.11) or wine intake (P interaction=0.97) (Additional file 1: Table S4) or between mucinous borderline tumors and total alcohol intake (data not shown). None of the other covariates statistically modified the alcohol- or wine-ovarian carcinoma association including race/ethnicity (P interactions ≥ 0.22) (data not shown).

## Discussion

While current guidelines for cancer prevention restrict alcohol drinking for women to no more than 1 drink per day [79], we found no evidence that recent moderate alcohol drinking increased overall ovarian carcinoma risk. Although there was some indication of effect modification by cell type, the statistical evidence was weak. This is the largest study to date to perform this evaluation quantitatively across the five types of ovarian carcinomas and the two groups of borderline ovarian tumors using individual-level data on alcohol intake.

Various oncogenic mechanisms of alcohol are well documented [80]. Although the evidence is convincing that alcohol is a risk factor for cancers of the breast and several cancers of the gastrointestinal tract [81], it has been equivocal for ovarian cancer [17,26-33]. Overall, our investigation adds to the evidence that recent moderate alcohol consumption is not significantly associated with ovarian carcinoma. A number of design and analytic factors can lead to disparate findings across studies. For example, heterogeneity of risk estimates were observed in European studies, a finding also reported by others [38]. The type and range of alcohol intake varies considerably across European countries and should be interpreted carefully when data are pooled or evaluated meta-analytically across continents.

Previous studies reported decreased risk from wine intake [26,27,31,82], although most associations were not statistically significant. The most widely reported constituents in wine are the polyphenols, including resveratrol, which derive mainly from the aerial tissues (grape skin) because their biosynthesis is stimulated by light [83]. Numerous anticarcinogenic properties of the polyphenols have been proposed [84]. A potentially interesting finding in the current study is the association between higher recent intakes of wine with decreased risk of clear cell ovarian carcinoma. The association persisted following exclusion of the European studies. A decreased risk from wine, but not beer or liquor, intake was also found among women in a pooled analysis of 12 prospective studies of renal cell carcinoma [85]. Clear cell ovarian carcinomas share similar features with clear cell renal cell carcinomas [86-88] and it has been suggested that moderate alcohol intake may improve insulin sensitivity and regulate related pathways [73,74] that are implicated in the etiology of these carcinomas [75-78]. We observed suggestive decreased risks of wine intake among non-obese women for clear cell and high-grade serous ovarian carcinomas; however, we cannot exclude the possibility that these findings are due to chance.

Several key non-genetic risk factors for ovarian cancer were reported in the early 1990s, when studies established decreased risks associated with oral contraceptive use [10,11], parity [12,13] and breast-feeding [12]. Using our consortium data, we recently reported that endometriosis was associated with increased risk of endometrioid and clear cell ovarian carcinomas [23]. However, few modifiable risk factors for ovarian cancers have been found. Perhaps the only lifestyle factor that is most consistently associated with modified risk of ovarian cancers is smoking, which is associated with an increased risk of both mucinous ovarian carcinoma and mucinous borderline tumors [24,25]. This emphasizes the importance of, and the need for more, pooled analyses of individual-level data that are harmonized carefully across different studies through collaborations within consortia, such as OCAC. Clearly, the research community struggles to understand the causes of the majority of these elusive and deadly cancers.

The strengths of this investigation include the analysis of individual-level data from a large sample as well as evaluation of higher levels of intakes (>3 drinks/d) and the standardized method of alcohol analysis, which allowed us to quantify risk associations based on average daily grams of alcohol intake. Although we attempted to reduce potential misclassification of histologic type by applying a pathology-based algorithm, the associations were not appreciably different if the algorithm was not implemented, unlike previous analyses for other exposures where implementation of the algorithm appeared to refine those associations [69]. The large sample of histologic types permitted evaluation of a wider range of alcohol intake, particularly total alcohol and wine intake. While a potential limitation of all case-control studies is recall and selection bias, our pooled estimates are in agreement with other reports [36,38]. Furthermore, although our findings relating moderate alcohol intake near the time of diagnosis indicated no association with ovarian carcinoma, it is possible that alcohol intake at other points in the life cycle may influence risk, given the long latency period estimated for these cancers [89].

## Conclusions

In conclusion, the results of this investigation do not support an association between recent moderate total alcohol intake and ovarian carcinoma overall. The findings do not strongly support variation in risk associated with specific histologic types. Understanding the modifiable causes of these deadly cancers through rigorous consortium analyses remains a priority for the research community.

## Competing interests

The authors declare that they have no competing interests.

## Authors’ contributions

LEK conceived the study design, performed the statistical analysis and drafted the manuscript; LEK, EVB, KLT, MAR, LAB, JAD, RBN, M Köbel, SHO, HY, PMW, JMS, MTG and HAR interpreted the data; EVB, KLT, MAR, LAB, JAD, RBN, SKK, JCC, GL, PJT, MEC, KM, RE, CB, AJ, EH, DWC, AFV, SHO, M King, UC, JL, MGC, HY, PMW, JMS, MTG and HAR coordinated contributing studies and provided data; All authors contributed to, and approved, the final manuscript version.

## Pre-publication history

The pre-publication history for this paper can be accessed here:

http://www.biomedcentral.com/1471-2407/13/28/prepub

## Supplementary Material

Additional file 1 **Table S1. **Alcohol intake distributions across study sites by case status, OCAC studies. **Table S2. **Distribution of covariates among cases and controls and among beer, wine and liquor consumers, OCAC studies. **Table S3. **Association between total alcohol and wine intake and histological types of ovarian carcinoma (original histological assignment), OCAC studies. **Table S4. **Association between total alcohol and wine intake and ovarian carcinoma, stratified by smoking, OCAC studies. Click here for file

## References

[B1] Ferlay J Shin HR Bray F Forman D Mathers C Parkin DM Estimates of worldwide burden of cancer in 2008: GLOBOCAN 2008 Int J Cancer 2010 127 12 2893 2917 10.1002/ijc.25516 21351269

[B2] Lynch HT Schuelke GS Kimberling WJ Albano WA Lynch JF Biscone KA Lipkin ML Deschner EE Mikol YB Sandberg AA Hereditary nonpolyposis colorectal cancer (Lynch syndromes I and II). II. Biomarker studies Cancer 1985 56 4 939 951 10.1002/1097-0142(19850815)56:4<939::AID-CNCR2820560440>3.0.CO;2-T 4016686

[B3] Lynch HT Conway T Lynch J Hereditary ovarian cancer. Pedigree studies, Part II Canc Genet Cytogenet 1991 53 2 161 183 10.1016/0165-4608(91)90094-B 2065292

[B4] Boyd J Rubin SC Hereditary ovarian cancer: molecular genetics and clinical implications Gynecol Oncol 1997 64 2 196 206 10.1006/gyno.1996.4572 9038264

[B5] Narod SA Madlensky L Bradley L Cole D Tonin P Rosen B Risch HA Hereditary and familial ovarian cancer in southern Ontario Cancer 1994 74 8 2341 2346 10.1002/1097-0142(19941015)74:8<2341::AID-CNCR2820740819>3.0.CO;2-Z 7922985

[B6] Risch HA McLaughlin JR Cole DE Rosen B Bradley L Kwan E Jack E Vesprini DJ Kuperstein G Abrahamson JL Prevalence and penetrance of germline BRCA1 and BRCA2 mutations in a population series of 649 women with ovarian cancer Am J Hum Genet 2001 68 3 700 710 10.1086/318787 11179017 PMC1274482

[B7] Song H Ramus SJ Tyrer J Bolton KL Gentry-Maharaj A Wozniak E Anton-Culver H Chang-Claude J Cramer DW DiCioccio R A genome-wide association study identifies a new ovarian cancer susceptibility locus on 9p22.2 Nat Genet 2009 41 9 996 1000 10.1038/ng.424 19648919 PMC2844110

[B8] Goode EL Chenevix-Trench G Song H Ramus SJ Notaridou M Lawrenson K Widschwendter M Vierkant RA Larson MC Kjaer SK A genome-wide association study identifies susceptibility loci for ovarian cancer at 2q31 and 8q24 Nat Genet 2010 42 10 874 879 10.1038/ng.668 20852632 PMC3020231

[B9] Bolton KL Tyrer J Song H Ramus SJ Notaridou M Jones C Sher T Gentry-Maharaj A Wozniak E Tsai YY Common variants at 19p13 are associated with susceptibility to ovarian cancer Nat Genet 2010 42 10 880 884 10.1038/ng.666 20852633 PMC3125495

[B10] Hankinson SE Colditz GA Hunter DJ Spencer TL Rosner B Stampfer MJ A quantitative assessment of oral contraceptive use and risk of ovarian cancer Obstet Gynecol 1992 80 4 708 714 1407899

[B11] The reduction in risk of ovarian cancer associated with oral-contraceptive use. The Cancer and Steroid Hormone Study of the Centers for Disease Control and the National Institute of Child Health and Human Development N Engl J Med 1987 316 11 650 655 3821795 10.1056/NEJM198703123161102

[B12] Whittemore AS Harris R Itnyre J Characteristics relating to ovarian cancer risk: collaborative analysis of 12 US case-control studies. II. Invasive epithelial ovarian cancers in white women. Collaborative ovarian cancer group Am J Epidemiol 1992 136 10 1184 1203 1476141 10.1093/oxfordjournals.aje.a116427

[B13] Risch HA Marrett LD Howe GR Parity, contraception, infertility, and the risk of epithelial ovarian cancer Am J Epidemiol 1994 140 7 585 597 7942759 10.1093/oxfordjournals.aje.a117296

[B14] Kurian AW Balise RR McGuire V Whittemore AS Histologic types of epithelial ovarian cancer: have they different risk factors? Gynecol Oncol 2005 96 2 520 530 10.1016/j.ygyno.2004.10.037 15661246

[B15] Gertig DM Hunter DJ Cramer DW Colditz GA Speizer FE Willett WC Hankinson SE Prospective study of talc use and ovarian cancer J Natl Canc Inst 2000 92 3 249 252 10.1093/jnci/92.3.249 10655442

[B16] Huncharek M Geschwind JF Kupelnick B Perineal application of cosmetic talc and risk of invasive epithelial ovarian cancer: a meta-analysis of 11,933 subjects from sixteen observational studies Anticancer Res 2003 23 2C 1955 1960 12820486

[B17] Kelemen LE Sellers TA Vierkant RA Harnack L Cerhan JR Association of folate and alcohol with risk of ovarian cancer in a prospective study of postmenopausal women Canc Causes Contr 2004 15 10 1085 1093 10.1007/s10552-004-1546-6 15801492

[B18] Genkinger JM Hunter DJ Spiegelman D Anderson KE Arslan A Beeson WL Buring JE Fraser GE Freudenheim JL Goldbohm RA Dairy products and ovarian cancer: a pooled analysis of 12 cohort studies Canc Epidemiol Biomarkers Prev 2006 15 2 364 372 10.1158/1055-9965.EPI-05-0484 16492930

[B19] Kushi LH Mink PJ Folsom AR Anderson KE Zheng W Lazovich D Sellers TA Prospective study of diet and ovarian cancer Am J Epidemiol 1999 149 1 21 31 10.1093/oxfordjournals.aje.a009723 9883790

[B20] Fairfield KM Hankinson SE Rosner BA Hunter DJ Colditz GA Willett WC Risk of ovarian carcinoma and consumption of vitamins A, C, and E and specific carotenoids: a prospective analysis Cancer 2001 92 9 2318 2326 10.1002/1097-0142(20011101)92:9<2318::AID-CNCR1578>3.0.CO;2-7 11745286

[B21] Schulz M Lahmann PH Boeing H Hoffmann K Allen N Key TJ Bingham S Wirfalt E Berglund G Lundin E Fruit and vegetable consumption and risk of epithelial ovarian cancer: the European Prospective Investigation into Cancer and Nutrition Canc Epidemiol Biomarkers Prev 2005 14 11 Pt 1 2531 2535 10.1158/1055-9965.EPI-05-0159 16284374

[B22] Risch HA Marrett LD Jain M Howe GR Differences in risk factors for epithelial ovarian cancer by histologic type. Results of a case-control study Am J Epidemiol 1996 144 4 363 372 10.1093/oxfordjournals.aje.a008937 8712193

[B23] Pearce CL Templeman C Rossing MA Lee A Near AM Webb PM Nagle CM Doherty JA Cushing-Haugen KL Wicklund KG Association between endometriosis and risk of histological subtypes of ovarian cancer: a pooled analysis of case-control studies Lancet Oncol 2012 13 4 385 394 10.1016/S1470-2045(11)70404-1 22361336 PMC3664011

[B24] Rossing MA Cushing-Haugen KL Wicklund KG Weiss NS Cigarette smoking and risk of epithelial ovarian cancer Canc Causes Contr 2008 19 4 413 420 10.1007/s10552-007-9103-8 18080774

[B25] Green A Purdie D Bain C Siskind V Webb PM Cigarette smoking and risk of epithelial ovarian cancer (Australia) Canc Causes Contr 2001 12 8 713 719 10.1023/A:1011297403819 11562111

[B26] Gwinn ML Webster LA Lee NC Layde PM Rubin GL Alcohol consumption and ovarian cancer risk Am J Epidemiol 1986 123 5 759 766 3962959 10.1093/oxfordjournals.aje.a114304

[B27] Webb PM Purdie DM Bain CJ Green AC Alcohol, wine, and risk of epithelial ovarian cancer.[see comment] Canc Epidemiol Biomarkers Prev 2004 13 4 592 599 15066924

[B28] Riman T Dickman PW Nilsson S Nordlinder H Magnusson CM Persson IR Some life-style factors and the risk of invasive epithelial ovarian cancer in Swedish women Eur J Epidemiol 2004 19 11 1011 1019 10.1007/s10654-004-1633-8 15648594

[B29] Chang ET Canchola AJ Lee VS Clarke CA Purdie DM Reynolds P Bernstein L Stram DO Anton-Culver H Deapen D Wine and other alcohol consumption and risk of ovarian cancer in the California Teachers Study cohort Canc Causes Contr 2007 18 1 91 103 10.1007/s10552-006-0083-x PMC1764867 17186425

[B30] Peterson NB Trentham-Dietz A Newcomb PA Chen Z Hampton JM Willett WC Egan KM Alcohol consumption and ovarian cancer risk in a population-based case-control study Int J Cancer 2006 119 10 2423 2427 10.1002/ijc.22137 16921486

[B31] Tworoger SS Gertig DM Gates MA Hecht JL Hankinson SE Caffeine, alcohol, smoking, and the risk of incident epithelial ovarian cancer Cancer 2008 112 5 1169 1177 10.1002/cncr.23275 18213613

[B32] La Vecchia C Negri E Franceschi S Parazzini F Gentile A Fasoli M Alcohol and epithelial ovarian cancer J Clin Epidemiol 1992 45 9 1025 1030 10.1016/0895-4356(92)90119-8 1432017

[B33] Hartge P Schiffman MH Hoover R McGowan L Lesher L Norris HJ A case-control study of epithelial ovarian cancer Am J Obstet Gynecol 1989 161 1 10 16 2750791 10.1016/0002-9378(89)90221-4

[B34] Modugno F Ness RB Allen GO Alcohol consumption and the risk of mucinous and nonmucinous epithelial ovarian cancer Obstet Gynecol 2003 102 6 1336 1343 10.1016/j.obstetgynecol.2003.08.008 14662224

[B35] Kuper H Titus-Ernstoff L Harlow BL Cramer DW Population based study of coffee, alcohol and tobacco use and risk of ovarian cancer Int J Cancer 2000 88 2 313 318 10.1002/1097-0215(20001015)88:2<313::AID-IJC26>3.0.CO;2-5 11004686

[B36] Genkinger JM Hunter DJ Spiegelman D Anderson KE Buring JE Freudenheim JL Goldbohm RA Harnack L Hankinson SE Larsson SC Alcohol intake and ovarian cancer risk: a pooled analysis of 10 cohort studies Br J Cancer 2006 94 5 757 762 16495916 10.1038/sj.bjc.6603020 PMC2361197

[B37] Kelemen LE Goode EL Alcohol intake increases risk of mucinous epithelial ovarian cancer: a meta-analysis Am J Epidemiol 2007 165 suppl S89 Abstract #355

[B38] Rota M Pasquali E Scotti L Pelucchi C Tramacere I Islami F Negri E Boffetta P Bellocco R Corrao G Alcohol drinking and epithelial ovarian cancer risk. A systematic review and meta-analysis Gynecol Oncol 2012 125 3 758 763 10.1016/j.ygyno.2012.03.031 22449732

[B39] Schouten LJ Zeegers MP Goldbohm RA van den Brandt PA Alcohol and ovarian cancer risk: results from the Netherlands Cohort Study Canc Causes Contr 2004 15 2 201 209 10.1023/B:CACO.0000019512.71560.2b 15017133

[B40] Phillips CV Publication bias in situ BMC Med Res Methodol 2004 4 20 10.1186/1471-2288-4-20 15296515 PMC514545

[B41] Kobel M Kalloger SE Boyd N McKinney S Mehl E Palmer C Leung S Bowen NJ Ionescu DN Rajput A Ovarian carcinoma subtypes are different diseases: implications for biomarker studies PLoS Med 2008 5 12 e232 10.1371/journal.pmed.0050232 19053170 PMC2592352

[B42] Wong KK Gershenson D The continuum of serous tumors of low malignant potential and low-grade serous carcinomas of the ovary Dis Markers 2007 23 5–6 377 387 18057521 10.1155/2007/204715 PMC3850577

[B43] Risch HA Bale AE Beck PA Zheng W PGR +331 A/G and increased risk of epithelial ovarian cancer Canc Epidemiol Biomarkers Prev 2006 15 9 1738 1741 10.1158/1055-9965.EPI-06-0272 16985038

[B44] Rossing MA Cushing-Haugen KL Wicklund KG Doherty JA Weiss NS Menopausal hormone therapy and risk of epithelial ovarian cancer Canc Epidemiol Biomarkers Prev 2007 16 12 2548 2556 10.1158/1055-9965.EPI-07-0550 18086757

[B45] Goodman MT Lurie G Thompson PJ McDuffie KE Carney ME Association of two common single-nucleotide polymorphisms in the CYP19A1 locus and ovarian cancer risk Endocr Relat Canc 2008 15 4 1055 1060 10.1677/ERC-08-0104 PMC2663409 18667686

[B46] Ness RB Dodge RC Edwards RP Baker JA Moysich KB Contraception methods, beyond oral contraceptives and tubal ligation, and risk of ovarian cancer Ann Epidemiol 2011 21 3 188 196 10.1016/j.annepidem.2010.10.002 21109450 PMC3052991

[B47] Schildkraut JM Iversen ES Wilson MA Clyde MA Moorman PG Palmieri RT Whitaker R Bentley RC Marks JR Berchuck A Association between DNA damage response and repair genes and risk of invasive serous ovarian cancer PLoS One 2010 5 4 e10061 10.1371/journal.pone.0010061 20386703 PMC2851649

[B48] Schildkraut JM Moorman PG Bland AE Halabi S Calingaert B Whitaker R Lee PS Elkins-Williams T Bentley RC Marks JR Cyclin E overexpression in epithelial ovarian cancer characterizes an etiologic subgroup Canc Epidemiol Biomarkers Prev 2008 17 3 585 593 10.1158/1055-9965.EPI-07-0596 18349276

[B49] Terry KL De Vivo I Titus-Ernstoff L Shih MC Cramer DW Androgen receptor cytosine, adenine, guanine repeats, and haplotypes in relation to ovarian cancer risk Cancer Res 2005 65 13 5974 5981 10.1158/0008-5472.CAN-04-3885 15994977 PMC1364476

[B50] Terry KL Tworoger SS Goode EL Gates MA Titus-Ernstoff L Kelemen LE Sellers TA Hankinson SE Cramer DW MTHFR polymorphisms in relation to ovarian cancer risk Gynecol Oncol 2010 119 2 319 324 10.1016/j.ygyno.2010.08.007 20817226 PMC2952716

[B51] Bandera EV King M Chandran U Paddock LE Rodriguez-Rodriguez L Olson SH Phytoestrogen consumption from foods and supplements and epithelial ovarian cancer risk: a population-based case control study BMC Womens Health 2011 11 40 10.1186/1472-6874-11-40 21943063 PMC3196697

[B52] Chandran U Bandera EV Williams-King MG Paddock LE Rodriguez-Rodriguez L Lu SE Faulkner S Pulick K Olson SH Healthy eating index and ovarian cancer risk Canc Causes Contr 2011 22 4 563 571 10.1007/s10552-011-9728-5 PMC3131161 21286802

[B53] Risch HA Jain M Marrett LD Howe GR Dietary fat intake and risk of epithelial ovarian cancer J Natl Canc Inst 1994 86 18 1409 1415 10.1093/jnci/86.18.1409 8072035

[B54] Royar J Becher H Chang-Claude J Low-dose oral contraceptives: protective effect on ovarian cancer risk Int J Cancer 2001 95 6 370 374 10.1002/1097-0215(20011120)95:6<370::AID-IJC1065>3.0.CO;2-T 11668519

[B55] Glud E Kjaer SK Thomsen BL Hogdall C Christensen L Hogdall E Bock JE Blaakaer J Hormone therapy and the impact of estrogen intake on the risk of ovarian cancer Arch Intern Med 2004 164 20 2253 2259 10.1001/archinte.164.20.2253 15534163

[B56] Huusom LD Frederiksen K Hogdall EV Glud E Christensen L Hogdall CK Blaakaer J Kjaer SK Association of reproductive factors, oral contraceptive use and selected lifestyle factors with the risk of ovarian borderline tumors: a Danish case-control study Canc Causes Contr 2006 17 6 821 829 10.1007/s10552-006-0022-x 16783610

[B57] Soegaard M Jensen A Hogdall E Christensen L Hogdall C Blaakaer J Kjaer SK Different risk factor profiles for mucinous and nonmucinous ovarian cancer: results from the Danish MALOVA study Canc Epidemiol Biomarkers Prev 2007 16 6 1160 1166 10.1158/1055-9965.EPI-07-0089 17548679

[B58] Garcia-Closas M Brinton LA Lissowska J Richesson D Sherman ME Szeszenia-Dabrowska N Peplonska B Welch R Yeager M Zatonski W Ovarian cancer risk and common variation in the sex hormone-binding globulin gene: a population-based case-control study BMC Canc 2007 7 60 10.1186/1471-2407-7-60 PMC1855931 17411440

[B59] Merritt MA Green AC Nagle CM Webb PM Talcum powder, chronic pelvic inflammation and NSAIDs in relation to risk of epithelial ovarian cancer Int J Cancer 2008 122 1 170 176 10.1002/ijc.23017 17721999

[B60] Ibiebele TI Parekh S Mallitt KA Hughes MC O'Rourke PK Webb PM Reproducibility of food and nutrient intake estimates using a semi-quantitative FFQ in Australian adults Publ Health Nutr 2009 12 12 2359 2365 10.1017/S1368980009005023 19257921

[B61] Patterson RE Kristal AR Tinker LF Carter RA Bolton MP Agurs-Collins T Measurement characteristics of the Women's Health Initiative food frequency questionnaire Ann Epidemiol 1999 9 3 178 187 10.1016/S1047-2797(98)00055-6 10192650

[B62] Hankin JH Wilkens LR Kolonel LN Yoshizawa CN Validation of a quantitative diet history method in Hawaii Am J Epidemiol 1991 133 6 616 628 2006649 10.1093/oxfordjournals.aje.a115934

[B63] Willett W Sampson L Stampfer MJ Rosner B Bain C Witschi J Hennekens CH Speizer FE Reproducibility and validity of a semiquantitative food frequency questionnaire Am J Epidemiol 1985 122 1 51 65 4014201 10.1093/oxfordjournals.aje.a114086

[B64] Rothman KJ Greenland S Modern epidemiology 1998 2 Philadelphia, PA: Lippincott-Reaven Publishers

[B65] Higgins JP Thompson SG Quantifying heterogeneity in a meta-analysis Stat Med 2002 21 11 1539 1558 10.1002/sim.1186 12111919

[B66] Smith-Warner SA Spiegelman D Ritz J Albanes D Beeson WL Bernstein L Berrino F van den Brandt PA Buring JE Cho E Methods for pooling results of epidemiologic studies: the pooling project of prospective studies of diet and cancer Am J Epidemiol 2006 163 11 1053 1064 10.1093/aje/kwj127 16624970

[B67] Willett W Stampfer MJ Total energy intake: implications for epidemiologic analyses Am J Epidemiol 1986 124 1 17 27 3521261 10.1093/oxfordjournals.aje.a114366

[B68] Hosmer DW Lemeshow SL Applied logistic regression 2000 New York, NY: John Wiley and Sons, Inc

[B69] Kelemen LE Goodman MT McGuire V Rossing MA Webb PM Kobel M Anton-Culver H Beesley J Berchuck A Brar S Genetic variation in TYMS in the one-carbon transfer pathway is associated with ovarian carcinoma types in the Ovarian Cancer Association Consortium Canc Epidemiol Biomarkers Prev 2010 19 7 1822 1830 10.1158/1055-9965.EPI-09-1317 PMC3013232 20570913

[B70] Seidman JD Kurman RJ Ronnett BM Primary and metastatic mucinous adenocarcinomas in the ovaries: incidence in routine practice with a new approach to improve intraoperative diagnosis Am J Surg Pathol 2003 27 7 985 993 10.1097/00000478-200307000-00014 12826891

[B71] Kobel M Kalloger SE Huntsman DG Santos JL Swenerton KD Seidman JD Gilks CB Differences in tumor type in low-stage versus high-stage ovarian carcinomas Int J Gynecol Pathol 2010 29 3 203 211 10.1097/PGP.0b013e3181c042b6 20407318

[B72] Gilks CB Ionescu DN Kalloger SE Kobel M Irving J Clarke B Santos J Le N Moravan V Swenerton K Tumor cell type can be reproducibly diagnosed and is of independent prognostic significance in patients with maximally debulked ovarian carcinoma Hum Pathol 2008 39 8 1239 1251 10.1016/j.humpath.2008.01.003 18602670

[B73] Davies MJ Baer DJ Judd JT Brown ED Campbell WS Taylor PR Effects of moderate alcohol intake on fasting insulin and glucose concentrations and insulin sensitivity in postmenopausal women: a randomized controlled trial JAMA 2002 287 19 2559 2562 10.1001/jama.287.19.2559 12020337

[B74] Calle EE Kaaks R Overweight, obesity and cancer: epidemiological evidence and proposed mechanisms Nat Rev Cancer 2004 4 8 579 591 15286738 10.1038/nrc1408

[B75] Brokaw J Katsaros D Wiley A Lu L Su D Sochirca O de la Longrais IA Mayne S Risch H Yu H IGF-I in epithelial ovarian cancer and its role in disease progression Growth Factors 2007 25 5 346 354 10.1080/08977190701838402 18236213

[B76] Kobel M Xu H Bourne PA Spaulding BO Shih Ie M Mao TL Soslow RA Ewanowich CA Kalloger SE Mehl E IGF2BP3 (IMP3) expression is a marker of unfavorable prognosis in ovarian carcinoma of clear cell subtype Mod Pathol 2009 22 3 469 475 10.1038/modpathol.2008.206 19136932

[B77] Parker AS Cheville JC Janney CA Cerhan JR High expression levels of insulin-like growth factor-I receptor predict poor survival among women with clear-cell renal cell carcinomas Hum Pathol 2002 33 8 801 805 10.1053/hupa.2002.126186 12203212

[B78] Rosendahl AH Holly JM Celander M Forsberg G Systemic IGF-I administration stimulates the in vivo growth of early, but not advanced, renal cell carcinoma Int J Cancer 2008 123 6 1286 1291 10.1002/ijc.23642 18561321

[B79] Kushi LH Doyle C McCullough M Rock CL Demark-Wahnefried W Bandera EV Gapstur S Patel AV Andrews K Gansler T American Cancer Society Guidelines on nutrition and physical activity for cancer prevention: reducing the risk of cancer with healthy food choices and physical activity CA Cancer J Clin 2012 62 1 30 67 10.3322/caac.20140 22237782

[B80] Seitz HK Stickel F Molecular mechanisms of alcohol-mediated carcinogenesis Nat Rev Cancer 2007 7 8 599 612 17646865 10.1038/nrc2191

[B81] World Cancer Research Fund/American Institute for Cancer Research Food, nutrition, physical activity, and the prevention of cancer: a global perspective 2007 Washington, DC: AICR

[B82] Goodman MT Tung KH Alcohol consumption and the risk of borderline and invasive ovarian cancer Obstet Gynecol 2003 101 6 1221 1228 10.1016/S0029-7844(03)00050-4 12798528

[B83] Manach C Scalbert A Morand C Remesy C Jimenez L Polyphenols: food sources and bioavailability Am J Clin Nutr 2004 79 5 727 747 15113710 10.1093/ajcn/79.5.727

[B84] Scalbert A Manach C Morand C Remesy C Jimenez L Dietary polyphenols and the prevention of diseases Crit Rev Food Sci Nutr 2005 45 4 287 306 10.1080/1040869059096 16047496

[B85] Lee JE Hunter DJ Spiegelman D Adami HO Albanes D Bernstein L van den Brandt PA Buring JE Cho E Folsom AR Alcohol intake and renal cell cancer in a pooled analysis of 12 prospective studies J Natl Canc Inst 2007 99 10 801 810 10.1093/jnci/djk181 17505075

[B86] Gavallos G Tawfik O Herrell D Langenstroer P Renal-ovarian axis: a case report and review Urology 2003 62 4 749 14550464 10.1016/s0090-4295(03)00585-5

[B87] Matsumura N Mandai M Okamoto T Yamaguchi K Yamamura S Oura T Baba T Hamanishi J Kang HS Matsui S Sorafenib efficacy in ovarian clear cell carcinoma revealed by transcriptome profiling Canc Sci 2010 101 12 2658 2663 10.1111/j.1349-7006.2010.01736.x PMC11159119 21040214

[B88] Zorn KK Bonome T Gangi L Chandramouli GV Awtrey CS Gardner GJ Barrett JC Boyd J Birrer MJ Gene expression profiles of serous, endometrioid, and clear cell subtypes of ovarian and endometrial cancer Clin Cancer Res 2005 11 18 6422 6430 10.1158/1078-0432.CCR-05-0508 16166416

[B89] Risch HA Hormone replacement therapy and the risk of ovarian cancer Gynecol Oncol 2002 86 2 115 117 10.1006/gyno.2002.6774 12144814

