# Supplementary material for: Recent alcohol consumption and risk of incident ovarian carcinoma: a pooled analysis of 5,342 cases and 10,358 controls from the Ovarian Cancer Association Consortium
Source: BMC Cancer. 2013 Jan 22;13:28. doi: 10.1186/1471-2407-13-28 (PMC3568733; doi:10.1186/1471-2407-13-28)
Supplement: Additional file 1 — Table S1. Alcohol intake distributions across study sites by case status, OCAC studies. Table S2. Distribution of covariates among cases and controls and among beer, wine and liquor consumers, OCAC studies. Table S3. Association between total alcohol and wine intake and histological types of ovarian carcinoma (original histological assignment), OCAC studies. Table S4. Association between total alcohol and wine intake and ovarian carcinoma, stratified by smoking, OCAC studies. [file 1471-2407-13-28-S1.doc]

**Supplementary Table 1.** Alcohol intake distributions across study sites by case status, OCAC studies

| **Study** | **Case status*** | **Total alcohol intake (gr/d) among drinkers†** | | | | **% alcohol drinkers‡** | **% beer drinkers§** | **% wine drinkers§** | **% liquor drinkers§** |
| --- | --- | --- | --- | --- | --- | --- | --- | --- | --- |
|  |  | 25^th^ percentile | 50% percentile | 75^th^ percentile | 95^th^ percentile |  |  |  |  |
| All studies | Cases | 1.10 | 4.52 | 12.5 | 37.7 | 57 | 25 | 47 | 33 |
|  | Controls | 1.54 | 5.48 | 13.8 | 49.2 | 58 | 28 | 49 | 34 |
| AUS | Cases | 0.97 | 4.62 | 10.2 | 31.5 | 74 | 29 | 62 | 49 |
|  | Controls | 1.27 | 5.44 | 11.6 | 31.2 | 82 | 32 | 72 | 58 |
| CON | Cases | 0.34 | 1.82 | 6.07 | 21.2 | 79 | 25 | 66 | 44 |
|  | Controls | 0.58 | 2.99 | 7.86 | 18.6 | 82 | 32 | 70 | 51 |
| DOV | Cases | 1.71 | 7.17 | 18.7 | 49.1 | 56 | 23 | 45 | 28 |
|  | Controls | 2.22 | 7.29 | 18.2 | 47.6 | 60 | 25 | 50 | 30 |
| GER | Cases | 3.0 | 5.0 | 19.0 | 48.0 | 56 | 23 | 49 | 11 |
|  | Controls | 3.0 | 7.0 | 14.0 | 38.0 | 73 | 32 | 67 | 14 |
| HAW | Cases | 13.1 | 33.0 | 96.3 | 268 | 28 | 13 | 21 | 10 |
|  | Controls | 12.4 | 38.0 | 111 | 289 | 38 | 16 | 30 | 16 |
| HOP | Cases | 12.2 | 14.2 | 28.4 | 54.8 | 16 | 9 | 3 | 6 |
|  | Controls | 12.2 | 14.2 | 35.2 | 87.7 | 15 | 8 | 4 | 7 |
| MAL | Cases | 1.29 | 4.83 | 11.2 | 27.4 | 91 | 69 | 88 | 70 |
|  | Controls | 1.58 | 5.17 | 10.8 | 34.3 | 92 | 68 | 87 | 68 |
| NCO | Cases | 0.41 | 2.38 | 9.43 | 29.2 | 60 | 26 | 51 | 34 |
|  | Controls | 0.41 | 2.24 | 8.87 | 28.2 | 60 | 29 | 48 | 35 |
| NEC | Cases | 1.81 | 5.40 | 12.3 | 34.5 | 65 | 23 | 53 | 31 |
|  | Controls | 1.51 | 4.64 | 11.1 | 33.1 | 70 | 32 | 57 | 30 |
| NJO | Cases | 0.36 | 1.53 | 6.50 | 19.4 | 77 | 39 | 67 | 52 |
|  | Controls | 0.53 | 2.18 | 7.15 | 15.4 | 78 | 36 | 73 | 54 |
| POL | Cases | 1.53 | 2.40 | 5.73 | 14.0 | 32 | 16 | 27 | 28 |
|  | Controls | 1.20 | 2.80 | 5.33 | 16.0 | 29 | 16 | 23 | 27 |
| SON | Cases | 1.71 | 5.67 | 15.1 | 35.8 | 56 | 21 | 41 | 28 |
|  | Controls | 1.44 | 4.72 | 13.4 | 35.9 | 55 | 20 | 38 | 29 |

*Ovarian carcinomas only

†Drinkers defined as any alcohol intake

‡Any type of alcohol (beer, wine or liquor)

§Percentages are not mutually exclusive between beer, wine and liquor drinkers

**Supplementary Table 2.** Distribution of covariates among cases and controls and among beer, wine and liquor consumers, OCAC studies

| **Variable** | **Level** | **Cases** | | **Controls** | | **Controls: Drinkers** | | |
| --- | --- | --- | --- | --- | --- | --- | --- | --- |
|  |  |  |  |  |  | **Beer** | **Wine** | **Liquor** |
|  |  | N | % | N | % | % | % | % |
| Age, years | <40 | 348 | 7 | 1,022 | 10 | 15 | 11 | 11 |
|  | 40-49 | 1,097 | 20 | 2,283 | 22 | 27 | 23 | 23 |
|  | 50-59 | 1,692 | 32 | 3,122 | 30 | 29 | 31 | 31 |
|  | 60-69 | 1,450 | 27 | 2,524 | 24 | 19 | 24 | 23 |
|  | ≥70 | 755 | 14 | 1,407 | 14 | 9 | 11 | 11 |
| Smoking, n | Never | 2,839 | 53 | 5,433 | 52 | 43 | 50 | 46 |
|  | Current | 939 | 18 | 2,106 | 20 | 26 | 20 | 24 |
|  | Former | 1,557 | 29 | 2,814 | 27 | 31 | 31 | 30 |
|  | Unknown or missing | 7 | <1 | 5 | <1 | <1 | <1 | <1 |
| Ethnicity, n | White non-Hispanic | 4,722 | 88 | 9,052 | 87 | 92 | 93 | 93 |
|  | White Hispanic | 27 | <1 | 64 | <1 | <1 | <1 | <1 |
|  | Black non-Hispanic | 150 | 3 | 284 | 3 | 2 | 2 | 2 |
|  | Asian | 216 | 4 | 426 | 4 | 2 | 2 | 1 |
|  | Other or unknown or missing | 227 | 4 | 532 | 5 | 4 | 3 | 3 |
| Menopausal status, n | Pre/peri-menopause | 1,485 | 28 | 3,637 | 35 | 45 | 37 | 38 |
|  | Post-menopause | 3,811 | 71 | 6,575 | 63 | 53 | 62 | 61 |
|  | Unknown or missing | 46 | <1 | 146 | 1 | 1 | 1 | <1 |
| Oral contraceptive use, months | < 6 | 2,782 | 52 | 4,177 | 40 | 32 | 33 | 33 |
|  | 6 - 22 | 588 | 11 | 1,091 | 11 | 10 | 10 | 10 |
|  | ≥ 23 | 1,913 | 36 | 5,030 | 49 | 57 | 56 | 56 |
|  | Unknown or missing | 59 | 1 | 60 | <1 | <1 | <1 | <1 |
| Tubal ligation, n | No | 4,387 | 82 | 7,887 | 76 | 80 | 79 | 78 |
|  | Yes | 926 | 17 | 2,439 | 24 | 20 | 20 | 22 |
|  | Unknown or missing | 29 | <1 | 32 | <1 | <1 | <1 | <1 |
| Endometriosis, n | No | 4,805 | 90 | 9,741 | 94 | 94 | 94 | 94 |
|  | Yes | 476 | 9 | 564 | 5 | 5 | 5 | 5 |
|  | Unknown or missing | 61 | 1 | 53 | <1 | <1 | <1 | <1 |
| Hysterectomy, n | No | 4,051 | 76 | 8,812 | 85 | 88 | 86 | 87 |
|  | Yes | 1,103 | 21 | 1,538 | 15 | 12 | 14 | 13 |
|  | Unknown or missing | 188 | 4 | 8 | <1 | <1 | <1 | <1 |
| Family history* | No | 1,490 | 28 | 3,342 | 32 | 34 | 35 | 35 |
|  | Yes | 423 | 8 | 627 | 6 | 6 | 7 | 6 |
|  | No daughters or sisters | 2,728 | 51 | 4,943 | 48 | 52 | 50 | 50 |
|  | Unknown or missing | 701 | 13 | 1,446 | 14 | 8 | 9 | 8 |
| Parity, n/Age at last pregnancy, yrs | Nulliparous | 1,088 | 20 | 1,271 | 12 | 15 | 13 | 13 |
|  | 1-2 births/ ≤25 years of age | 650 | 12 | 1,107 | 11 | 10 | 9 | 11 |
|  | ≥3 births/ ≤25 years of age | 206 | 4 | 394 | 4 | 3 | 3 | 3 |
|  | 1-2 births/ >25 years of age | 1,739 | 33 | 3,998 | 39 | 43 | 42 | 40 |
|  | ≥3 births/ >25 years of age | 1,465 | 27 | 3,421 | 33 | 28 | 31 | 31 |
|  | Pregnancy status or last age at pregnancy unknown or missing | 194 | 4 | 167 | 2 | 2 | 2 | 2 |
| Interview year | 1990-1994 | 612 | 11 | 830 | 8 | 7 | 7 | 7 |
|  | 1995-1999 | 828 | 15 | 2,392 | 23 | 34 | 31 | 27 |
|  | 2000-2004 | 2,591 | 48 | 4,819 | 46 | 44 | 48 | 50 |
|  | 2005-2009 | 1,309 | 25 | 2,316 | 22 | 14 | 14 | 16 |
|  | Unknown or missing | 2 | <1 | 1 | <1 |  | <1 |  |
| Age at menarche, yrs | 8 -10 | 313 | 6 | 585 | 6 | 4 | 4 | 4 |
|  | 11 | 724 | 14 | 1,311 | 13 | 11 | 12 | 12 |
|  | 12 | 1,287 | 24 | 2,447 | 24 | 24 | 24 | 23 |
|  | 13 | 1,444 | 27 | 2,771 | 27 | 27 | 28 | 29 |
|  | 14 - 21 | 1,530 | 29 | 3,166 | 31 | 33 | 31 | 31 |
|  | <8 or ≥22 or unknown or missing | 44 | <1 | 78 | 1 | <1 | <1 | <1 |
| BMI, kg/m^2^ | 15 - 18.4 | 106 | 2 | 219 | 2 | 2 | 3 | 2 |
|  | 18.5 - 24.9 | 2,489 | 46 | 5,088 | 49 | 57 | 55 | 52 |
|  | 25 - 29.9 | 1,477 | 28 | 2,927 | 28 | 27 | 27 | 28 |
|  | 30 - 34.9 | 681 | 13 | 1,238 | 12 | 8 | 10 | 10 |
|  | 35 - 39.9 | 292 | 5 | 470 | 5 | 3 | 3 | 4 |
|  | 40 - 49.9 | 184 | 3 | 281 | 3 | 1 | 1 | 2 |
|  | <15 or ≥50 | 113 | 2 | 135 | 1 | <1 | <1 | 1 |
|  | Unknown or missing | 77 | 1 | 78 | <1 |  |  |  |
| Education | Less than high school | 978 | 18 | 1,883 | 18 | 25 | 20 | 23 |
|  | High school | 1,383 | 26 | 2,592 | 25 | 20 | 19 | 19 |
|  | Some college | 1,163 | 22 | 2,312 | 22 | 20 | 23 | 23 |
|  | College graduate | 737 | 14 | 1,594 | 15 | 15 | 15 | 14 |
|  | Graduate | 657 | 12 | 1,307 | 13 | 16 | 17 | 14 |
|  | Missing | 424 | 8 | 670 | 7 | 5 | 6 | 6 |

*Family history of breast or ovarian cancer in first-degree relatives (mother, sister, daughter)

**Supplementary Table 3.** Association between total alcohol and wine intake and histological types* of ovarian carcinoma, OCAC studies

| **Intake/d** | **Controls**  **N=10,358** | **Serous**  **N=2,948** | | **Mucinous**  **N=361** | | **Endometrioid**  **N=885** | | **Clear Cell**  **N=501** | |  |
| --- | --- | --- | --- | --- | --- | --- | --- | --- | --- | --- |
|  | Co | Ca | OR (95% CI) | Ca | OR (95% CI) | Ca | OR (95% CI) | Ca | OR (95% CI) | *P* value† |
| **Total alcohol** ‡ |  |  |  |  |  |  |  |  |  |  |
| None | 4,296 | 1,204 | 1.0 (Ref) | 145 | 1.0 (Ref) | 378 | 1.0 (Ref) | 223 | 1.0 (Ref) |  |
| Up to 1 drink | 3,928 | 1,177 | 0.97 (0.87-1.09) | 142 | 1.07 (0.81-1.41) | 352 | 0.91 (0.76-1.09) | 188 | 0.84 (0.66-1.07) |  |
| 1-2 drinks | 1,112 | 326 | 1.03 (0.88-1.20) | 39 | 1.14 (0.77-1.70) | 86 | 0.93 (0.71-1.21) | 53 | 0.97 (0.69-1.37) |  |
| 2-3 drinks | 400 | 98 | 0.86 (0.67-1.10) | 18 | 1.49 (0.87-2.53) | 38 | 1.21 (0.83-1.75) | 17 | 0.96 (0.56-1.63) |  |
| >3 drinks | 622 | 143 | 0.98 (0.79-1.21) | 17 | 0.88 (0.51-1.50) | 31 | 0.70 (0.47-1.04) | 20 | 0.82 (0.50-1.34) |  |
| P trend |  |  | 0.61 |  | 0.67 |  | 0.29 |  | 0.50 | 0.77 |
| >2 drinks § | 1,022 | 241 | 0.92 (0.78-1.10) | 35 | 1.12 (0.74-1.67) | 69 | 0.91 (0.68-1.21) | 37 | 0.88 (0.60-1.30) |  |
| P trend § |  |  | 0.58 |  | 0.49 |  | 0.43 |  | 0.53 | 0.78 |
| **Wine** ¶ |  |  |  |  |  |  |  |  |  |  |
| None | 5,307 | 1,511 | 1.0 (Ref) | 190 | 1.0 (Ref) | 462 | 1.0 (Ref) | 272 | 1.0 (Ref) |  |
| Up to 4oz | 3,984 | 1,157 | 0.95 (0.85-1.05) | 148 | 1.08 (0.83-1.40) | 350 | 0.94 (0.79-1.14) | 195 | 0.87 (0.69-1.09) |  |
| 4-8oz | 522 | 152 | 1.00 (0.82-1.24) | 14 | 0.93 (0.53-1.66) | 45 | 1.10 (0.78-1.55) | 24 | 0.95 (0.60-1.50) |  |
| >8oz | 545 | 128 | 0.93 (0.74-1.16) | 9 | 0.60 (0.30-1.21) | 28 | 0.74 (0.49-1.12) | 10 | 0.43 (0.22-0.83) |  |
| P trend |  |  | 0.45 |  | 0.38 |  | 0.32 |  | 0.02 | 0.16 |
| >4oz § | 1,067 | 280 | 0.97 (0.82-1.14) | 23 | 0.77 (0.48-1.22) | 73 | 0.93 (0.70-1.23) | 34 | 0.70 (0.47-1.03) |  |
| P trend § |  |  | 0.46 |  | 0.60 |  | 0.47 |  | 0.05 | 0.36 |

Adjusted for age (<40; 40-49; 50-59; 60-69; 70+ years), smoking status (never, former, current), site (AUS, CON, DOV, GER, HAW, HOP, MAL, NCO, NEC, NJO, POL, SON), race/ethnicity (white nonHispanic; white Hispanic; black non Hispanic; Asian; other or unknown); menopausal status (pre/peri-menopausal; postmenopausal, unknown or missing), oral contraceptive use (<6mo, 6-22 mo, 23+ mo, unknown or missing), tubal ligation (yes; no; unknown or missing), endometriosis (yes; no; unknown or missing), hysterectomy (yes; no; unknown or missing), family history of breast or ovarian cancer in first-degree relatives (no; yes; unknown; no daughters or sisters), parity/age at last birth (nulliparous; 1-2 births/age ≤25 yrs at last pregnancy; 3+ births/age ≤25 yrs at last pregnancy; 1-2 births/age >25 years at last pregnancy; 3+ births/age >25 years at last pregnancy; yes if ever pregnant but unknown or missing age at last pregnancy age; no or unknown if ever pregnant and missing age at last pregnancy, interview year (1990-1994; 1995-1999; 2000-2004; 2005-2009; missing), age at menarche (8-10 yrs; 11 yrs; 12 yrs; 13 yrs; 14-21 yrs; <8 or ≥ 22yrs), body mass index (continuous) and education (less than high school, high school, some college, completed college or university, completed graduate or professional degree, missing).

* Based on original histological assignment obtained from each study.

† *P* for tumor heterogeneity derived from testing the trend variable for alcohol or wine intake in polytomous regression models with 4 df (see Statistical analysis).

‡ 1 drink = 10 grams ethanol.

§ Risk estimates and P trend values are from models that collapse the two highest intake categories.

¶ Models are also simultaneously adjusted for consumption of beer and liquor intake.

**Supplementary Table 4.** Association between total alcohol intake and wine intake and ovarian carcinoma, stratified by smoking, OCAC studies

|  | **Never smokers** | | **Current smokers** | | **Former smokers** | |
| --- | --- | --- | --- | --- | --- | --- |
| **Intake/d** | **Ca/Co** | **OR (95% CI)** | **Ca/Co** | **OR (95% CI)** | **Ca/Co** | **OR (95% CI)** |
| **Total Alcohol** * |  |  |  |  |  |  |
| None | 1,408/2,561 | 1.0 (Ref) | 320/744 | 1.0 (Ref) | 537/989 | 1.0 (Ref) |
| Up to 1 drink | 1,061/2,029 | 0.89 (0.78-1.02) | 381/841 | 0.89 (0.69-1.17) | 630/1,056 | 1.04 (0.85-1.28) |
| 1-2 drinks | 226/483 | 0.88 (0.71-1.08) | 116/250 | 1.05 (0.74-1.47) | 217/378 | 1.09 (0.85-1.40) |
| 2-3 drinks | 71/159 | 0.83 (0.60-1.15) | 51/93 | 1.31 (0.83-2.07) | 70/148 | 0.81 (0.56-1.17) |
| >3 drinks | 73/201 | 0.84 (0.60-1.17) | 71/178 | 0.97 (0.65-1.46) | 103/243 | 1.06 (0.78-1.45) |
| P trend |  | 0.07 |  | 0.56 |  | 0.99 |
| P interaction | 0.11 |  |  |  |  |  |
| **Wine** † |  |  |  |  |  |  |
| None | 1,587/2,931 | 1.0 (Ref) | 511/1,109 | 1.0 (Ref) | 719/1,265 | 1.0 (Ref) |
| Up to 4oz | 1,053/2,026 | 0.93 (0.81-1.06) | 351/807 | 0.81 (0.64-1.04) | 650/1,149 | 0.98 (0.82-1.19) |
| 4-8oz | 123/260 | 0.94 (0.72-1.22) | 40/92 | 0.96 (0.58-1.60) | 98/169 | 1.04 (0.74-1.45) |
| >8oz | 76/216 | 0.80 (0.58-1.09) | 37/98 | 0.67 (0.39-1.16) | 90/231 | 0.87 (0.63-1.21) |
| P trend |  | 0.13 |  | 0.11 |  | 0.56 |
| P interaction | 0.97 |  |  |  |  |  |

Adjusted for age (<40; 40-49; 50-59; 60-69; 70+ years), site (AUS, CON, DOV, GER, HAW, HOP, MAL, NCO, NEC, NJO, POL, SON), race/ethnicity (white nonHispanic; white Hispanic; black non Hispanic; Asian; other or unknown); menopausal status (pre/peri-menopausal; postmenopausal, unknown or missing), oral contraceptive use (<6mo, 6-22 mo, 23+ mo, unknown or missing), tubal ligation (yes; no; unknown or missing), endometriosis (yes; no; unknown or missing), hysterectomy (yes; no; unknown or missing), family history of breast or ovarian cancer in first-degree relatives (no; yes; unknown; no daughters or sisters), parity/age at last birth (nulliparous; 1-2 births/age ≤25 yrs at last pregnancy; 3+ births/age ≤25 yrs at last pregnancy; 1-2 births/age >25 years at last pregnancy; 3+ births/age >25 years at last pregnancy; yes if ever pregnant but unknown or missing age at last pregnancy age; no or unknown if ever pregnant and missing age at last pregnancy, interview year (1990-1994; 1995-1999; 2000-2004; 2005-2009; missing), age at menarche (8-10 yrs; 11 yrs; 12 yrs; 13 yrs; 14-21 yrs; <8 or ≥ 22yrs), body mass index (continuous) and education (less than high school, high school, some college, completed college or university, completed graduate or professional degree, missing). Wine consumption was additionally adjusted for other alcoholic beverage types. Models include interaction terms between site and each covariate except alcohol.

* 1 drink = 10 grams ethanol.

† Models are also simultaneously adjusted for consumption of beer and liquor intake.
